# Orbital ordering and magnetism in layered Perovskite Ruthenate Sr_2_RuO_4_

**DOI:** 10.1038/s41598-020-63415-8

**Published:** 2020-04-27

**Authors:** Hung-Lung Huang, Horng-Tay Jeng

**Affiliations:** 1 Department of Physics, National Tsing Hua University, Hsinchu, 30013 Taiwan; 2 Physics Division, National Center for Theoretical Sciences, Hsinchu, 30013 Taiwan; 3 Institute of Physics, Academia Sinica, Taipei, 11529 Taiwan

**Keywords:** Electronic properties and materials, Magnetic properties and materials, Electronic properties and materials, Magnetic properties and materials

## Abstract

Local density approximation plus on-site Coulomb interaction *U* electronic structure calculations reveal that layered perovskite oxide Sr_2_RuO_4_ exhibits the ferromagnetic (FM) half-metallic ground state, which is nearly degenerate with the antiferromagnetic (AFM) phase with a slightly higher total energy. The nearly degenerate FM/AFM total energies provide a reasonable explanation for the experimentally observed spin-fluctuation. In addition, a dumbbell-shape 4*d* − *t*_2*g*_ recombined *d*_*xz*_ − *d*_*yz*_ orbital ordering on the Ru sublattice is obtained owing to the on-site Coulomb interaction *U* associated with the elongated RuO_6_ octahedron local structure. The discovered orbital ordering is robust against the spin-orbit interaction as well as the surface terminations. Our findings unravel the on-site Coulomb correlation as the driving force of the Ru-4d orbital ordering as well as the inherent magnetic degeneracy.

## Introduction

Rich physical properties of transition metal oxides, such as electronic, magnetic, and transport properties, is largely connected with the orbital, charge, spin, and lattice degrees of freedom. It was proposed that orbital ordering is closely related to the magnetic and crystallographic lattice in perovskites manganites^1^. The orbital ordering (OO) states are usually found in localized 3d systems where the cooperative Jahn-Teller distortions are significant due to the strong hybridization between the 3d and O 2p electrons^2^. Evidences for 3d − ${t}_{2g}$ OO have been found in titanates^3,4^, vanadates^5–8^, and magnetite^9–11^, while 4d − ${t}_{2g}$ OO state is also proposed for relatively itinerant ruthenate SrRuO_3_^12^ and Ca_2_RuO_4_^13,14^. Direct observation of OO in La_0.5_Sr_1.5_MnO_3_ using soft x-ray diffraction measurement^15^ has been reported. Very recently, a first real-space orbital ordering image at the cobalt-terminated surface of the well-studied heavy fermion compound CeCoIn_5_ is observed by using high accuracy sub-atomic resolution STM^16^. First-principles analysis shows that these staggered ${d}_{xy}-{d}_{yz}$ orbital orders are triggered by the enhanced on-site Coulomb interaction at the surface. With the success of the real-space direct experimental measurements of orbital ordering images, researches along this direction are highly encouraged.

Layered perovskite Ruthenates exhibit interesting properties such as anomalous superconductivity, Fermi liquid behavior, large electronic specific heat, and heavy fermion behavior^17–22^. Among them, Sr_2_RuO_4_, discovered by Y. Maeno and H. Hashimoto *et al*. in 1994, is the first copper-free superconductor in layered perovskite^17^. It has a lower superconducting transition temperature (T_*c*_ ~ 1.5 K) than the prototype high T_*c*_ superconductors (e.g. (La, Ba)_2_CuO_4_^23^). It is generally believed that this superconducting state has a spin triplet state^24–26^, which may be chiral p-wave state^27^. This makes Sr_2_RuO_4_ a potential candidate for intrinsic topological superconductor^28^, though debates remain among different experiments^29^. Moreover, the p-wave superconductivity in Sr_2_RuO_4_ is closely related to the quasi-two-dimensional ferromagnetism^25^, exchange of the ferromagnetic spin fluctuations^26,30^, or strongly momentum-dependent entanglement of spin and orbital^31^. Meanwhile, surface ferromagnetism has been proposed in a previous combined ARPES with ab-initio study^32^. Whereas possible surface antiferromagnetic state is also reported^26^.

Sr_2_RuO_4_ crystallizes in the tetragonal (space group I4/*mmm*) perovskite structure with 2 f.u. per unit cell^33^. It contains SrO-RuO_2_-SrO sandwiched layers with a lateral shift between alternative layers. In this work, we study the basic electronic and magnetic properties of Sr_2_RuO_4_ including the magnetism, orbital ordering, correlation, pressure, and strain effect using first-principles calculations based on density functional theory. We find that Sr_2_RuO_4_ exhibits nearly degenerate ferromagnetic half-metallic ground state and antiferromagnetic state, which provides a reasonable origin of the experimentally observed spin-fluctuation. In addition, we discover the spin polarized dumbbell-shape $4d-{t}_{2g}$ recombined ${d}_{xz}$ − ${d}_{yz}$ orbital ordering on the Ru sublattice driven by the strongly correlated on-site Coulomb interaction *U* associated with the elongated RuO_6_ octahedron local structure.

## Results and Discussions

### Electronic and Magnetic properties of bulk Sr_2_RuO_4_

**Figure 1:**
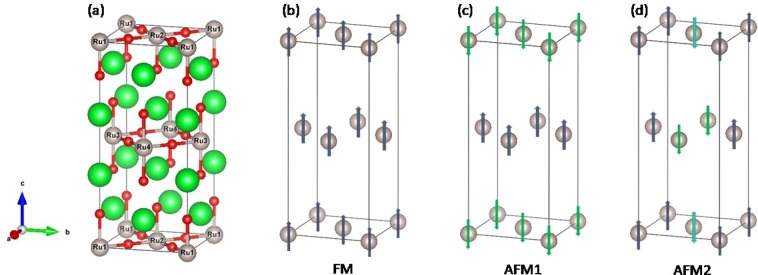
(**a**) Lattice structure of Sr_2_RuO_4_. The green, grey, and red spheres represent Sr, Ru, and O, respectively. (**b**–**d**) Three magnetic configuratios of Sr_2_RuO_4_: ferromagnetic (FM), antiferromagnetic 1 (AFM1) and 2 (AFM2), respectively. The nonmagnetic Sr and O ions are ignored in (**b**–**d**) to give a clear picture of the magnetic configurations in the Ru sublattice.

Figure 1(a) shows the tetragonal perovskite structural (I4/*mmm*) of Sr_2_RuO_4_ with 4 formula unit (f.u.) in the unit cell (u.c.). To study the magnetism based on this lattice structure, we consider the nonmagnetic (NM) phase and three possible magnetic configurations, i.e., ferromagnetic (FM), antiferromagnetic 1 (AFM1), and antiferromagnetic 2 (AFM2) as depicted in Fig. 1(b–d), respectively. Firstly we carry out geometry optimizations for the lattice structures of these four magnetic phases separately using different on-site *U* with/without SOC. Three conclusions can be reached based on the lattice relaxations: (I) Including or excluding the SOC has no obvious effect on the lattice structure. (II) With a fixed *U* value, the optimized lattice structures of different magnetic configurations are nearly the same. (III) Different $U$ values only slightly modify the lattice constants without making any other noticeable changes in the lattice structure. Therefore we only present the magnetic and electronic properties from $U=3.5$ eV and $J=0.6$ eV in the rest of this paper. The optimized lattice constants are $a=b=5.375$ Å and $c=12.338$ Å, being in good agreement with the experimental values of $a=b=5.46$ Å and $c=12.72$ Å, respectively.

Table 1 lists the total energy, total moment, and local moments of Ru ions from LDA + *U* calculations without and with spin-orbital coupling. Without taking SOC into account, the total energy of AFM1, AFM2 and NM phase is respectively 3.9, 62.6 and 154.3 meV/f.u. higher than that of the FM phase. Taking the SOC into consideration, the total energy of AFM1, AFM2 and NM is respectively 4.0, 61.4 and 152.8 meV/f.u. higher than FM. The relative stability of each magnetic structure from high to low is FM, AFM1, AFM2, and NM, no matter the SOC is considered or not. The FM ground state obtained here is consistent with the experimental observation of the quasi-two-dimensional ferromagnetism in Sr_2_RuO_4_^25^. The nearly degenerate AFM state with slightly higher total energy than the FM ground state also provides a reasonable explanation of the ferromagnetic spin fluctuations and possible surface antiferromagnetic state observed in experiments^26^.

**Table 1:** Total energy, total moment and local moments of Ru ions from LDA + *U* without and with spin-orbital coupling.

	ΔE (meV/u.c.)	Mag. (*μ*_*B*_/u.c.)	Ru1.mag (*μ*_*B*_/Ru)	Ru2.mag (*μ*_*B*_/Ru)	Ru3.mag (*μ*_*B*_/Ru)	Ru4.mag (*μ*_*B*_/Ru)
NM	617.3	0	0	0	0	0
FM	0.0	8	1.38	1.38	1.38	1.38
AFM1	15.5	0	−1.38	−1.38	1.38	1.38
AFM2	250.5	0	0.83	−0.79	0.79	−0.83
NM_*soc*_	611.0	(0, 0, 0)	(0, 0, 0)	(0, 0, 0)	(0, 0, 0)	(0, 0, 0)
FM_*soc*_	0.0	(0, 0, 7.96)	(0, 0, 1.38)	(0, 0, 1.38)	(0, 0, 1.38)	(0, 0, 1.38)
AFM1_*soc*_	16.1	(0, 0, 0)	(0, 0, −1.138)	(0, 0, −1.138)	(0, 0, 1.38)	(0, 0, 1.38)
AFM2_*soc*_	245.6	(0, 0, 0)	(0, 0, 0.8)	(0, 0, −0.79)	(0, 0, 0.79)	(0, 0, −0.8)

Our calculations show that Sr_2_RuO_4_ exhibits very similar total energies for the FM and AFM configurations with the difference of only 16 meV/u.c., or equivalently 4 meV/f.u. as listed in Table 1. This tiny difference actually indicates that the FM ground state is thermally not very stable and may easily turn into AFM with significant spin fluctuations at finite temperature. To estimate the Curie temperature (${T}_{C}$) of Sr_2_RuO_4_, we further calculate the exchange parameter $J$ based on our calculated total energies of different spin configurations through the Heisenberg model. The ${T}_{C}$ can be calculated by the mean-field approximation: ${T}_{C}=2J{S}^{2}/3{K}_{B}\approx 15K$. Such a low ${T}_{C}$ implies a strong spin fluctuation around 15 K. Further, the ${T}_{C}$ given by the mean-field approximation is usually overestimated, which means that the strong spin fluctuation should occur at a much lower temperature than 15 K. This picture is indeed compatible with the strong spin fluctuation picture demonstrated in previous experimental and theoretical investigations.

In fact, the long-standing debates on the spin fluctuation in Sr_2_RuO_4_ over the past 20 years is similar to what happened in the iron-based superconductor FeSe in the past 10 years. Theoretically, the AFM-II spin configuration is the ground state of FeSe. Nevertheless, magnetic signals have never been detected experimentally. Until recently, the polarized ultrafast spectroscopy finally confirmed that the intense and rapid spin fluctuation in FeSe may present non-magnetic state in general experiments^34^. Similar situations also happen in spin-polarized scanning tunnelling microscopy (SP-STM) measurements on magnetic materials such as Fe-thin film that thermal energy may drive the spin fluctuation and result in an averaged-out magnetic signal in SP-STM^35^. Even though some experiments and LDA + DMFT calculations^36^ suggest that Sr_2_RuO_4_ does not order magnetically, the scenario provided by the ultrafast spectroscopy for FeSe^34^ and SP-STM for Fe-thin film^35^ could help clarify the spin fluctuation issue in Sr_2_RuO_4_.

On the other hand, the total moment and local Ru moments remain almost the same as 1.38 *μ*_*B*_/Ru even if the SOC is included. These results show that in addition to the negligible effect on the lattice optimization from SOC, SOC not only has no obvious effect on the relative stability of the magnetic phases, but also SOC has no noticeable effect on the magnetic moments. Consequently one can conclude that the SOC in the Ru-4d orbital is not strong enough to make significant effects. Previous theoretical and experimental studies also show evidences of long range ferromagnetic order in Sr_2_RuO_4_ with the Ru magnetic moment ranging from ~0.2 to ~1.0 *μ*_*B*_^26,32,37,38^.

In the ionic model, the four 4d electrons of the Ru^4+^ ion occupy the ${t}_{2g}$ triplet leaving the higher ${e}_{g}$ doublet empty under the octahedral crystal field. In accordance with Hund’s rule, Ru^4+^ is in the high spin state with the spin alignment of (${t}_{2g}^{3\uparrow }$, ${t}_{2g}^{1\downarrow }$), giving rise to a moment of 2 *μ*_*B*_/Ru and a half-metallic ground state with the majority spin insulating and minority spin conducting^39^. Experimental and theoretical evidences of spin polarization enhanced by spin-triplet pairing have also been reported^40,41^. As shown in Table 1, our calculated total moment of Sr_2_RuO_4_ in the FM configuration from LDA + *U* agrees qualitatively with that given from the ionic model. However, the obtained moment 1.38 *μ*_*B*_ of Ru is somewhat smaller than that from the ionic model because of the significant *p*-*d* hybridization between the relatively extended Ru-4*d* and O-2*p* orbitals. There exists a nontrivial moment of 0.19 *μ*_*B*_ at the O_*planar*_ and 0.04 *μ*_*B*_ at the O_*apex*_.

For the antiferromagnetic configurations, Ru ions are divided into two sublattices with the spin moment of each Ru sublattice mutually antiparallel. For AFM1 (Fig. 1c), the spin moments of Ru1 and Ru2 in the same plane aligns ferromagnetically forming the spin up sublattice with Ru3 and Ru4 of the other plane forming the spin down sublattice. The magnitude of spin moments of the spin up (Ru1, Ru2) and spin down (Ru3, Ru4) sublattices are equal to that (1.38 *μ*_*B*_) of the FM configuration. While for AFM2 (Fig. 1d), the spin moments of Ru ions in the same plane align antiferromagnetically with each other, which is far different form AFM1 and FM phases. As a result, the magnitude of spin moments of the spin up (Ru1, Ru3) and spin down (Ru2, Ru4) sublattices are only 0.83 *μ*_*B*_ and 0.79 *μ*_*B*_, respectively.

**Figure 2:**
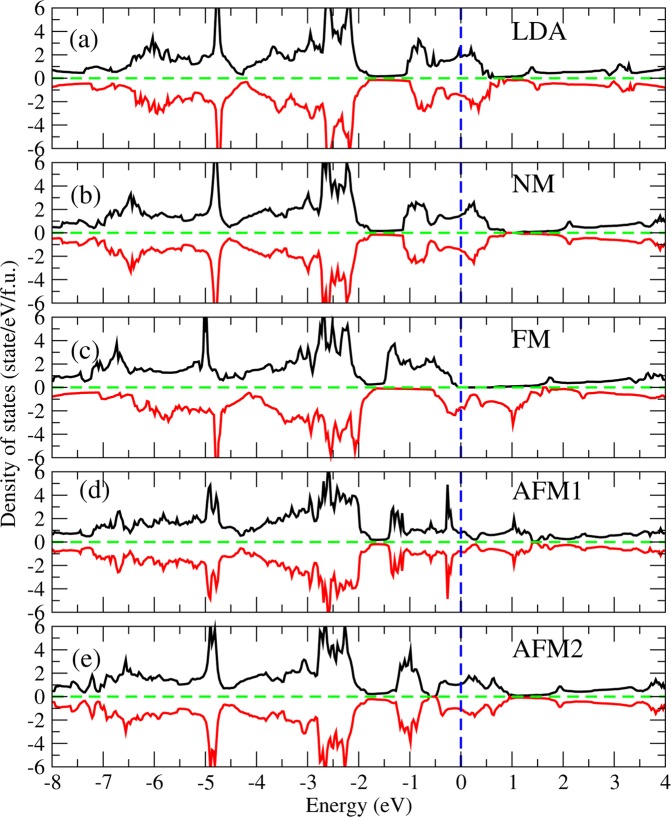
Density of states of Sr_2_RuO_4_ from (**a**) LDA(FM) and (**b**–**e**) LDA + U with 4 different magnetic configurations: NM, FM, AFM1, and AFM2. The Fermi level is at the zero energy.

Figure 2(a,b–e) show the density of states (DOS) of Sr_2_RuO_4_ from LDA and LDA + *U* calculations for NM, FM, AFM1, and AFM2 configurations, respectively. LDA gives a metallic ground state with the Ru ${t}_{2g}$ band distributed from 1 eV below to 0.5 eV above the Fermi level [Fig. 2(a)]. It also shows that the spin up and down DOS are approximately the same with a slight exchange splitting between the spin up and spin down channels. Therefore it is in the weak ferromagnetic ground state close to the nonmagnetic phase. Taking into account the on-site $U$, the Ru ${t}_{2g}$ band energy and bandwidth of the NM phase [Fig. 2(b)] are similar to those given by LDA [Fig. 2(a)].

For the FM phase shown in Fig. 2(c), the on-site *U* strongly enhances the exchange splitting of ~1 eV between the Ru ${t}_{2g}$ spin up ($[\,-\,1.6,-\,0.1]$ eV) and spin down ($[\,-\,0.6,1.4]$ eV) bands around the Fermi level, which is rather different from the weak ferromagnetism given from LDA [Fig. 2(a)]. Thus, the spin-up ${t}_{2g}$ band is fully occupied with the Fermi level lying in the enhanced energy gap, giving rise to the half-metallic ground state in consistency with the valence configuration (${t}_{2g}^{3\uparrow }$, ${t}_{2g}^{1\downarrow }$) of Ru from the ionic model. As for AFM1 and AFM2 [Fig. 2(d,e), respectively], the symmetric spin up and spin down DOS show zero total moment with large Ru local moment (Table 1). Owing to the laterally ferromagnetic while layer-to-layer antiferromagnetic nature of the Ru local moments, the Ru-${t}_{2g}$ bandwidth of AFM1 [Fig. 2(d)] is approximately from the lower bound of the spin up Ru-${t}_{2g}$ band to the upper bound of the spin down Ru-${t}_{2g}$ band in the FM phase [Fig. 2(c)]. On the contrary, the AFM2 phase [Fig. 2(e)] with the laterally antiferromagnetic nature, which has no common signature with the FM phase, shows suppressed bandwidth with strongly localized Ru-${t}_{2g}$ bands.

### Orbital ordering in bulk Sr_2_RuO_4_

**Figure 3:**
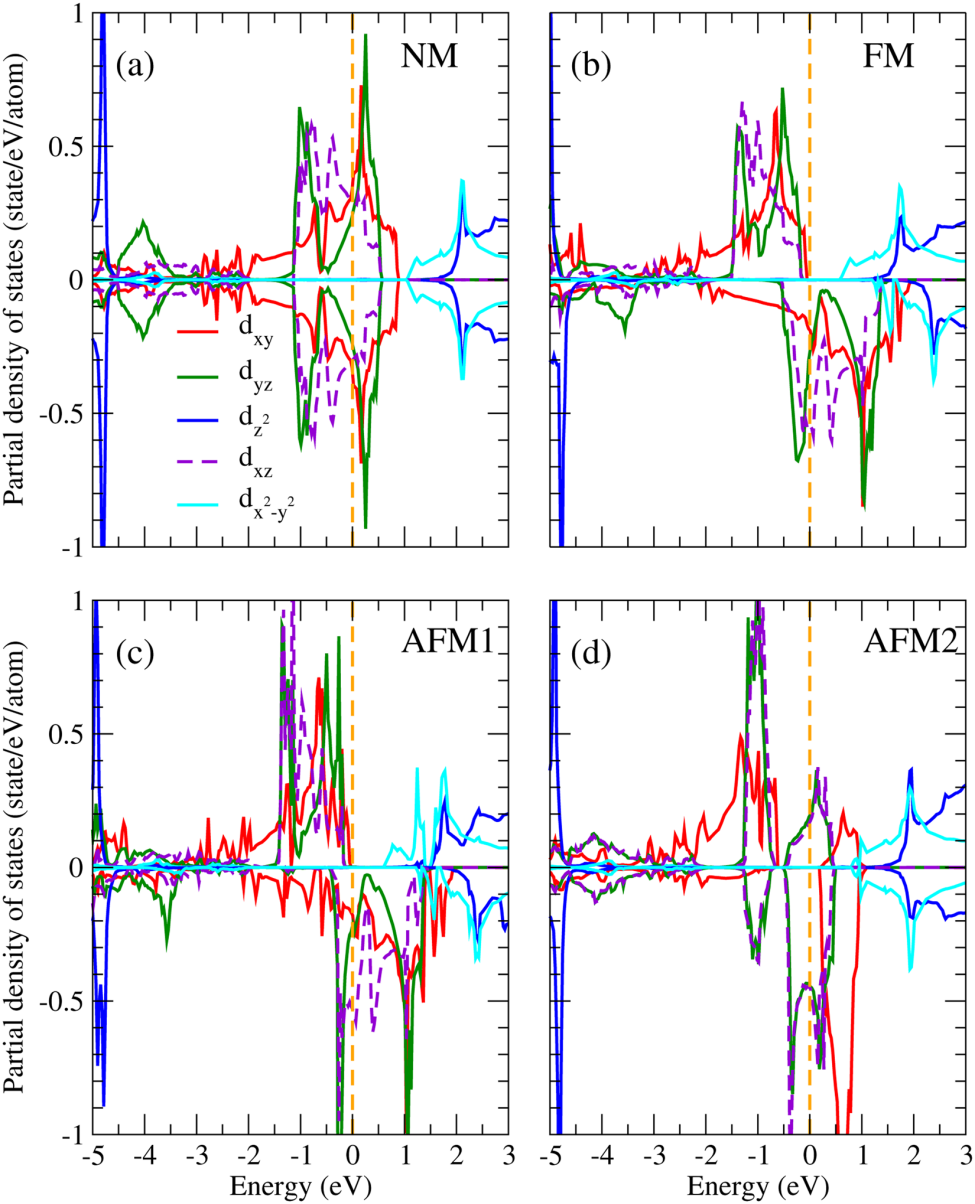
PDOS of Ru in Sr_2_RuO_4_ from LDA + *U* in the (**a**) non-magnetic, (**b**) ferromagnetic, (**c**) AFM1, and (**d**) AFM2 configurations.

To identify the orbital ordering state in Sr_2_RuO_4_, we present in Fig. 3 the partial DOS (PDOS) projected onto the five 4d orbitals of the Ru ion in the RuO_6_ octahedral local coordinates (*xyz*) with the $z$-axis directed to the crystal c axis and the $x$- and $y$- axes pointing to the crystal [110] and [1–10] directions, respectively. The NM PDOS in Fig. 3(a) shows that the three Ru-${t}_{2g}$ bands, i.e., ${d}_{xy}$, ${d}_{yz}$, and ${d}_{xz}$ significantly mixed up with each other in the vicinity of the Fermi level, therefore does not exhibit orbital ordering (OO). The FM (Fig. 3(b)) and AFM1 (Fig. 3(c)) PDOS are very similar: the majority spin channel opens up an energy gap of ~0.5 eV at the Fermi level with the three ${d}_{xy}$, ${d}_{yz}$, and ${d}_{xz}$ bands fully occupied below the Fermi level (−2.5~0.0 eV). In the minority spin channel, on the contrary, these three bands locate at the Fermi level in which the PDOS right below the Fermi level (−0.5~0.0 eV) mainly composed of the ${d}_{xz}$ and ${d}_{yz}$ orbitals with the ${d}_{xy}$ PDOS significantly suppressed. This is a clear sign of the ${d}_{xz}$ − ${d}_{yz}$ orbital ordering formation as will be further discussed later. The AFM2 PDOS in Fig. 3(d) shows different picture in which both the minority and majority PDOS around the Fermi level ([−0.5, 0.0] eV) are fully contributed by ${d}_{xz}$ and ${d}_{yz}$ orbitals. As a result, except NM, all the considered magnetic configurations FM, AFM1, and AFM2 exhibit OO behavior in the vicinity of the Fermi level. It is noted that previous optical conductivity and reflectivity measurements of Sr_2_RuO_4_ show strong anisotropy between the in-plane and out-of-plane spectra at room temperature^42^, which may stem from the orbital ordering discovered in this work.

**Figure 4:**
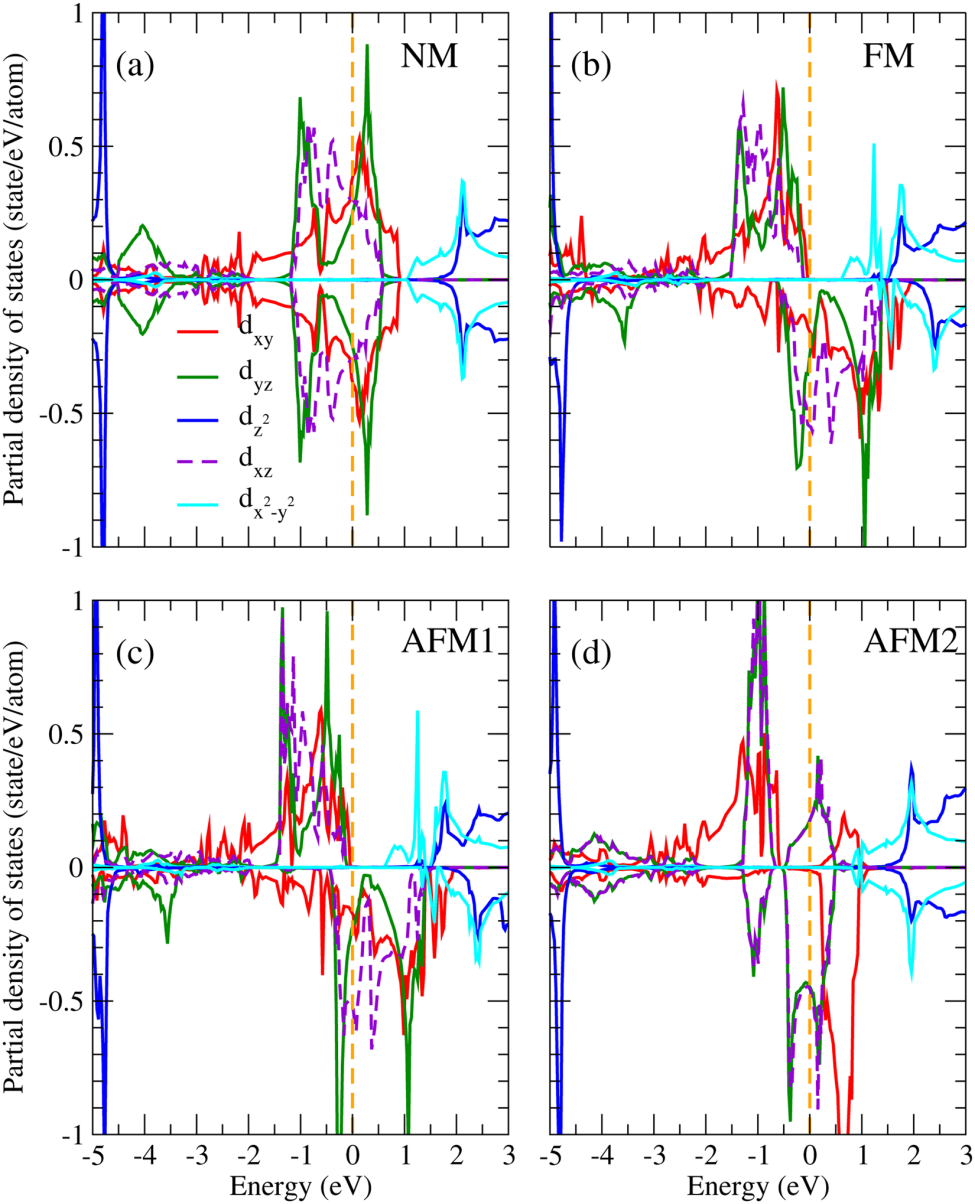
PDOS of Ru in Sr_2_RuO_4_ from LDA + *U* + SOC in the (**a**) nonmagnetic, (**b**) ferromagnetic, (**c**) AFM1, and (**d**) AFM2 configurations.

One significant issue is whether or not the spin-orbit coupling (SOC) would destroy the observed OO in Sr_2_RuO_4_. Without SOC, the cubic harmonics is a nature basis set in the octahedral crystal field and gives rise to the lower ${t}_{2g}$ and higher ${e}_{g}$ bands. However, the SOC, which couples the spin to the angular momentum of the charge distribution and hence the crystal structure, prefers the spherical harmonic basis set with the magnetic quantum numbers. Thus, the competitive SOC tends to mix the cubic harmonic basis set and could ruin the observed ${d}_{xz}$ − ${d}_{yz}$ OO. By including the SOC self-consistently in the LDA + *U* calculations, we found that in spite of the relatively strong SO coupling in 4*d* orbitals, the site and orbital decomposed PDOS shown in Fig. 4 as well as the OO pattern (not shown here) remain more or less the same as those in Fig. 3. The calculated total energy is 0.36 eV/Sr_2_RuO_4_ lower with a quenched orbital magnetic moment of 0.01 *μ*_*B*_/Ru. Similar trend has also been found in Ca_2_RuO_4_^43^ and SrRuO_3_^12^ that even with the SOC in 4d orbitals, the octahedral crystal field on Ru ions is strong enough to quench the orbital moment and to stabilize the OO. Thus, the obtained orbital ordered half-metallic ground state is robust upon varying $U$, lattice relaxation, and even upon including the SO interaction in the calculations.

**Figure 5:**
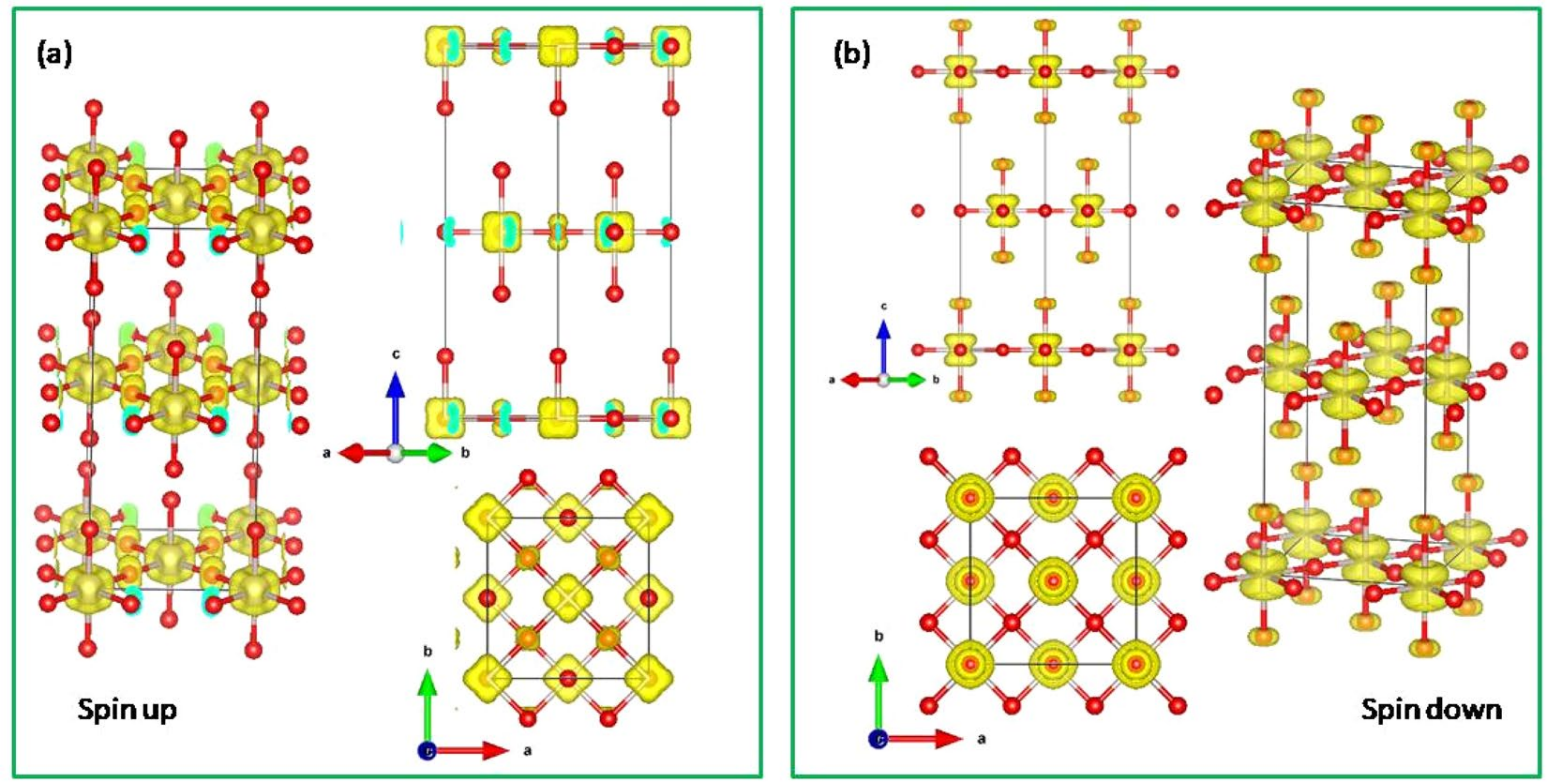
OO of Sr_2_RuO_4_ in the spin-up (**a**) and spin down (**b**) channels. Red and grey balls are O and Ru ions, respectively. Sr ions are omitted for a clear view.

Figure 5 illustrates the top, side, and perspective view of the spin up and spin down charge density isosurface corresponding to the FM ${t}_{2g}$ bands right below the Fermi level (−0.5~0.0 eV) [Fig. 3(b)]. The orbital ordering pattern is clearly seen that there exists a cube-like [Fig. 5(a)] and a dumbbell-like [Fig. 5(b)] charge density distribution on each Ru ion in the spin up and spin down channel, respectively. The spin up cube-like electron cloud reflects the fully occupied Ru-${t}_{2g}$ band in the spin up channel. While in the spin down channel, the dumbbell-like orbital ordering comes from the recombined Ru-${t}_{2g}$ ${d}_{xz}$ and ${d}_{yz}$ orbitals near the Fermi level [Fig. 3(b)]. Here the reasons for the spin polarized orbital ordering formation are very similar to those of SrRuO_3_^12^. In SrRuO_3_, the OO is given from the on-site $U$ associated with the Jahn-Teller distortion, in which the RuO_6_ octahedron distorts in the crystal $ab$ plane and rotates with zigzag tilting along the $c$ axis. Whereas in Sr_2_RuO_4_, the lattice structure is more symmetric than the SrRuO_3_ one that the RuO_6_ octahedron only elongated along the $c$-axis (Ru-O(*ab*-plane) = 1.9 Å, Ru-O(*c*-axis) = 2.0 Å) with a rotation over the $ab$ plane due to the tetragonal “layer-like” lattice structure of Sr_2_RuO_4_ [Fig. 1(a)]. With the Hubbard $U$ effect and the elongated RuO_6_ octahedron local structure simultaneously taken into account, Sr_2_RuO_4_ thus shows the ${d}_{xz}$ − ${d}_{yz}$ OO in the half-metallic ground state. This is why the dumbbell-shape OO pattern in Sr_2_RuO_4_ is more isotropic in the $ab$ plane than the cross-like OO pattern in SrRuO_3_. The total energy of this orbital ordering phase is ~50 meV/f.u. lower than that of the ideal structure. On the other hand, the geometry optimization and the lattice distortion have no prominent influence on the magnetic phases, indicating that the magnetism is not sensitive to the lattice deformation or the crystal field effect in Sr_2_RuO_4_.

### Orbital ordering in Sr_2_RuO_4_ surface

**Figure 6:**
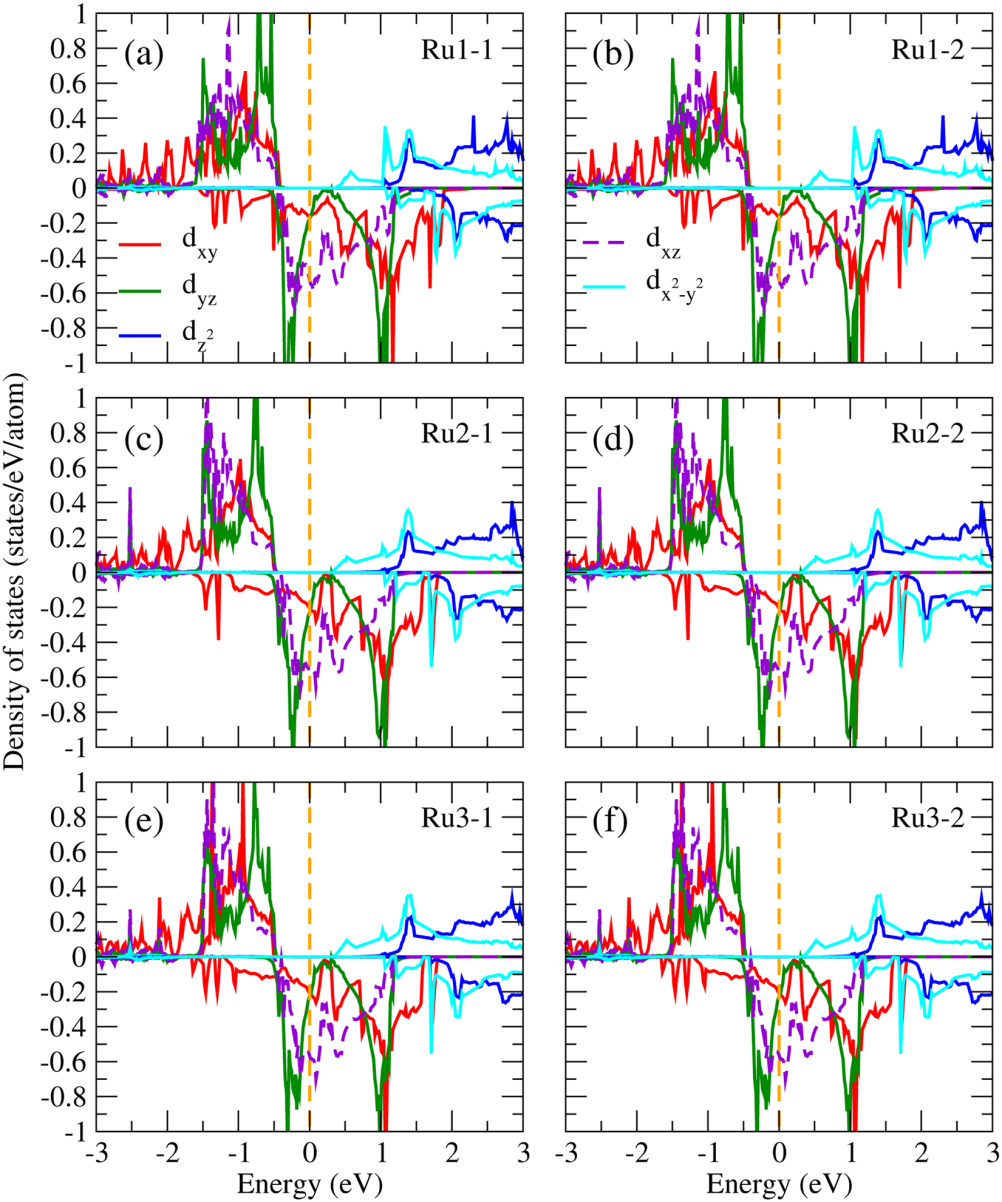
LDA + *U* PDOS of Ru in the optimized Sr_2_RuO_4_ slab structure with the SrO termination. Ru1, Ru2, and Ru3 indicate the first (surface), second, and third RuO_2_ layer, respectively. Two Ru ions in each layer are depicted in the left and right panels.

**Figure 7:**
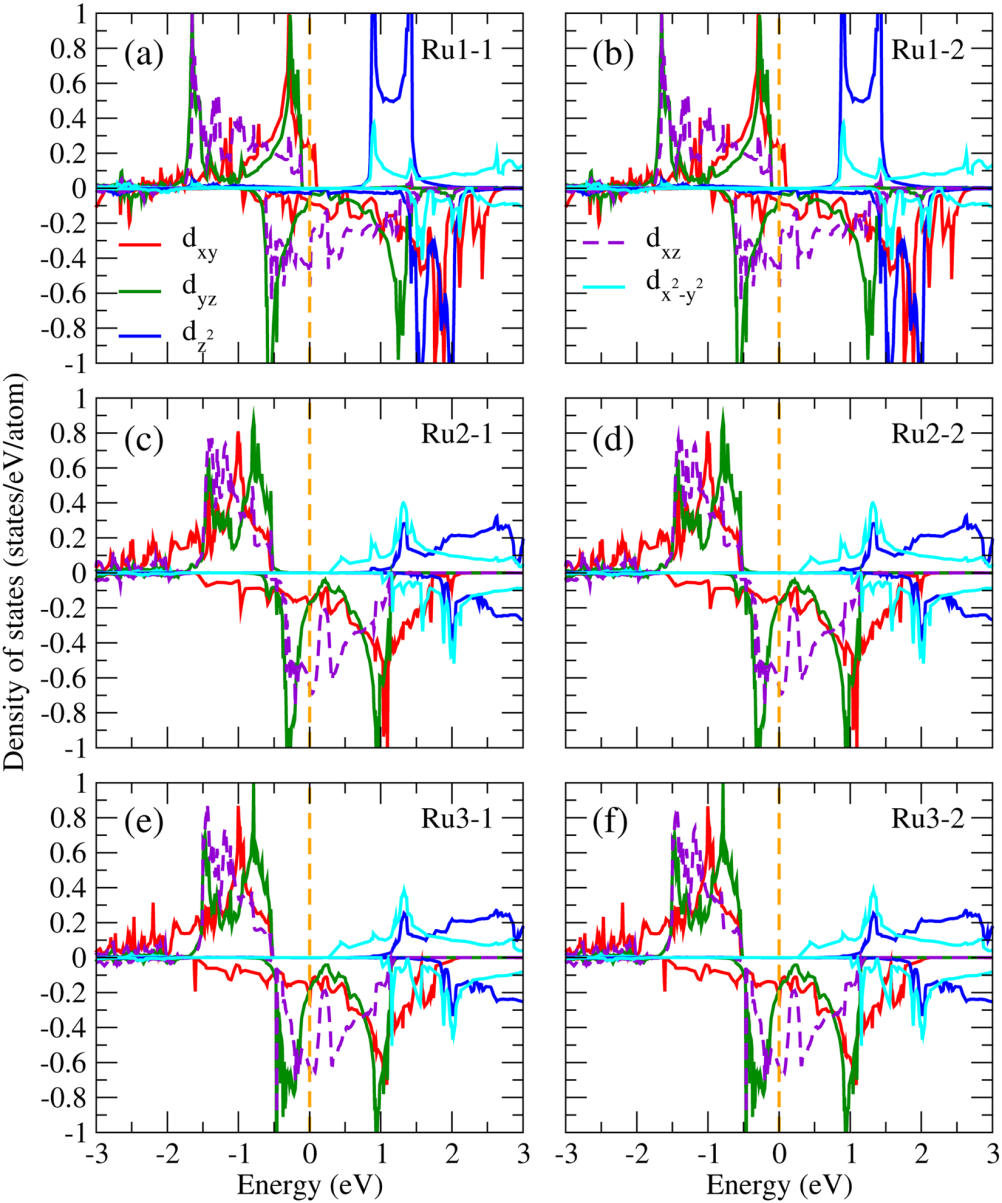
LDA + *U* PDOS of Ru in the relaxed Sr_2_RuO_4_ slab with the RuO_2_ terminated surface layers. Ru1, Ru2, and Ru3 indicate the first (surface), second, and third RuO_2_ layer, respectively. Two Ru ions in each layer are depicted in the left and right panels.

**Figure 8:**
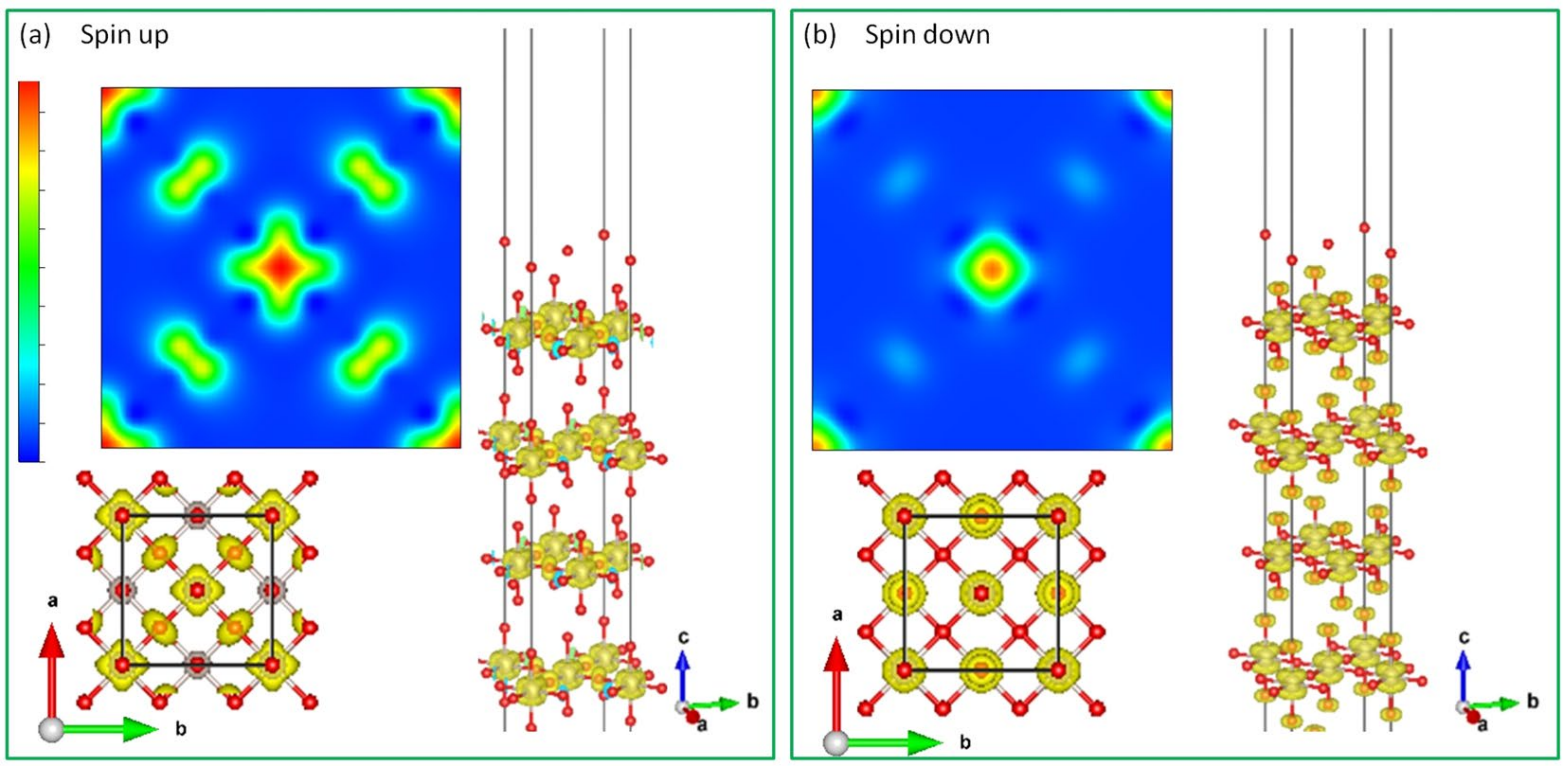
OO of Sr_2_RuO_4_ slab with SrO terminated surface layers. Red and grey balls are O and Ru ions, respectively. Sr ions are omitted for a clear view.

**Figure 9:**
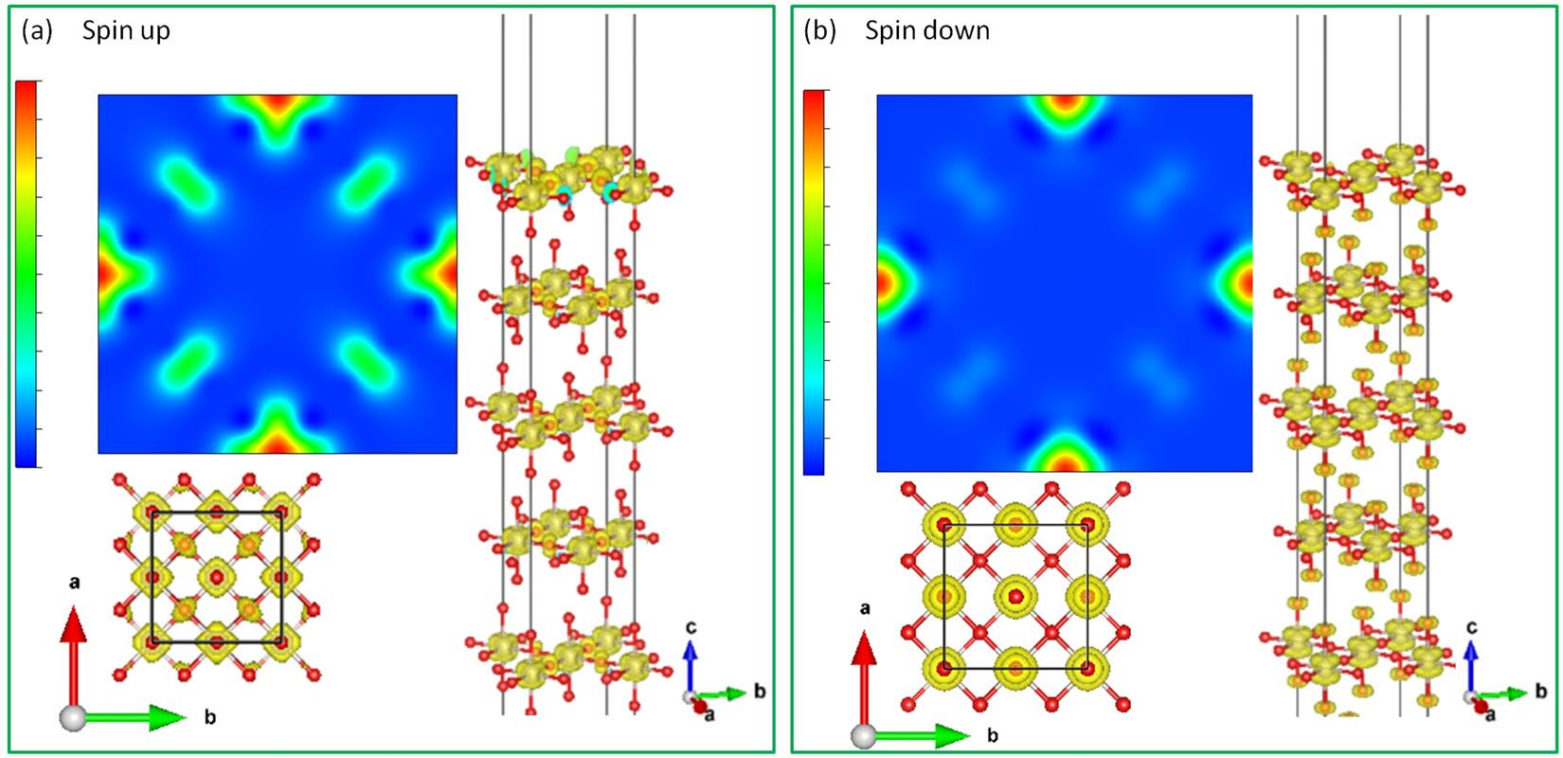
OO of Sr_2_RuO_4_ slab with RuO_2_ terminated surface layers. Red and grey balls are O and Ru ions, respectively. Sr ions are omitted for a clear view.

To understand the surface effect on the orbital ordering of Sr_2_RuO_4_, we also performed LDA + *U* slab calculations with lattice relaxation for both the SrO and RuO_2_ terminated surfaces as shown in Figs. 6 and 7, respectively. For the SrO-termination (Fig. 6), the electronic structures are similar to those of the bulk phase (Fig. 3(b)). This is presumably due to the fully ionic characters of the Sr^2+^ and O^2−^ at the SrO surface layer, and therefore the relatively stable close shell states. In contrast, for the RuO_2_-terminated case shown in Fig. 7, there exist significant deviations in the PDOS of the surface layer Ru ions (Fig. 7(a,b)) from those of the bulk phase in Fig. 3(b). Associated with the outwards displacement (0.21 Å) of the RuO_2_ surface layer, one of the unoccupied spin up ${e}_{g}$ bands, i.e., the ${d}_{{z}^{2}}$ band is energetically lowered by ~1.3 eV with the bandwidth reduced by ~1.5 eV [Fig. 7(a,b)]. This nontrivial band energy lowering and bandwidth suppression are due to the octahedral symmetry breaking at the surface Ru in the absence of the topmost apical O. In addition, the spin-down ${d}_{xy}$ band PDOS of the surface Ru ions (Fig. 7(a,b)) below the Fermi level is significantly suppressed also because of the missing topmost apical O and hence the preferred ${d}_{xz}$ and ${d}_{yz}$ orbital with reduced Coulomb repulsion from the O anion. The orbital ordering pattern at the SrO and RuO_2_ surface layer of Sr_2_RuO_4_ are presented in Figs. 8 and 9, respectively. We note that despite the variation in the PDOS of the surface Ru ions, overall, the observed orbital ordering in the bulk state (Fig. 5) remains more or less the same at the surfaces [Figs. 8 and 9]. We note that the real space direct evidence of orbital ordering formation at the CeCoIn_5_ surface has been observed by high-accuracy sub-atomic resolution STM experiments^16^. We hope our prediction of the orbital ordering in Sr_2_RuO_4_ bulk and surface can be verified by similar direct measurements in the near future.

### Effects of pressure and strain in bulk Sr_2_RuO_4_

**Figure 10:**
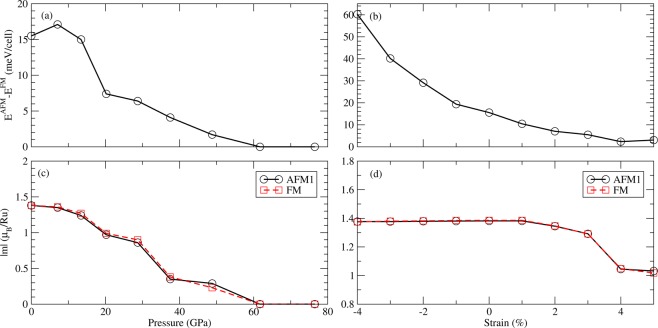
Total enery difference between FM and AFM states of Sr_2_RuO_4_ under isotropic pressure (**a**) and uniaxial strain (along z-axis) (**b**). Magnetic moment of Ru in FM phase (red) and AFM1 phase (black) under pressure (**c**) and strain (**d**).

The energy difference between AFM1 and FM phases, and the magnetic moments of Ru ion for AFM1 and FM phases versus pressure are presented in Fig. 10. As the pressure increases, the energy difference between AFM1 and FM, and the magnetic moments in Ru atom both decrease significantly. Based on the Heisenberg model and mean-field approximation, we also estimate the Curie temperature T_*c*_ of Sr_2_RuO_4_ under pressure. We found that when the pressure increases to 20 GPa, the energy difference between FM and AFM1 decreases to 7.4 meV. The corresponding T_*c*_ ~ 7 K is lower than the one without pressure. This indicates the spin fluctuations become stronger under pressure. Further increasing the pressure to 62 GPa, both FM and AFM1 states no longer exist. Both turn into the nonmagnetic phase.

**Figure 11:**
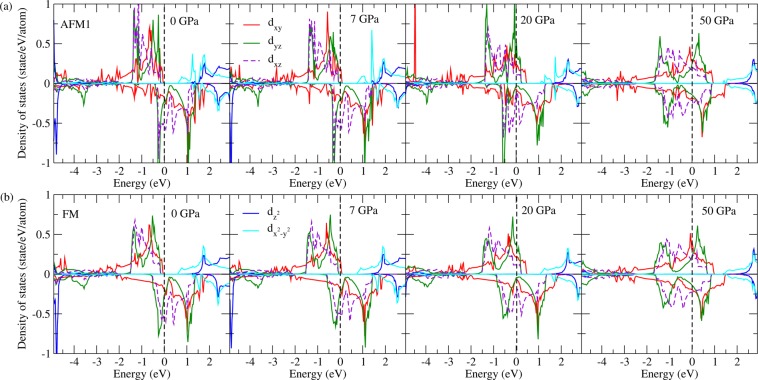
Evolution of Ru-PDOS along with pressure in the AFM (**a**) and FM (**b**) phases.

The evolution of PDOS of AFM1 and FM phases along with pressure are shown in Fig. 11. As the pressure increases, the energy distribution of the $4d-{t}_{2g}$ orbitals gradually shift to higher energies for the spin-up channel, and to lower energies for the spin-down channel, suppressing the exchange splitting significantly. The half-metallicity is ruined at 7 GPa and the magtism is terminated at 62 GPa. Similar behavior is also found in the FM phase. Pressure not only removes the half-metallicity and the mangetism but also suppresses the orbital ordering. As shown, the three $4d-{t}_{2g}$ bands are occuped more evenly under higher pressures, thus weakening the orbital ordering significantly.

On the other hand, we also take the uniaxial strain (along z-axis) effect into consideration. The energy difference between AFM1 and FM phases, and the magnetic moments of Ru ion for AFM1 and FM phases versus strain from −4% to 5% are shown in Fig. 10. The energy difference of E(FM)-E(AFM1) varies smoothly along with strain. When the compressive strain increases, the energy difference becomes larger. As the tensile strain increases, the energy difference is reduced. These results demonstrate that the tensile strain can strengthen the spin fluctuation and lower Tc, whereas the compressive strain behaves in the opposite way.

## Conclusions

In conclusion, the electronic and magnetic features of Sr_2_RuO_4_ have been systematically studied by means of LDA + *U* calculations. The calculated ferromagnetic half-metallic ground state is consistent with previous theoretical predictions as well as the simple ionic model and Hund’s rules. Moreover, we discover that Sr_2_RuO_4_ exhibits spin up $4d-{t}_{2g}$ and spin down ${d}_{xz}$ − ${d}_{yz}$ recombined dumbbell-shape orbital ordering in the bulk phase as well as at the surface. On the other hand, we find that the antiferromagnetic state (AFM1) of Sr_2_RuO_4_ with slightly higher energy also shows similar OO near the Fermi level as the FM state. These interesting electronic and magnetic behaviors exist only when the on-site *U* is considered, indicating the important role the strong correlations play in Sr_2_RuO_4_. Our finding unravels the nature of the OO, the magnetism, the on-site Coulomb repulsion $U$ in the relatively extended Ru-4*d* orbitals, and the correlations among them in Sr_2_RuO_4_. Furthermore, we discover that isotropic pressure and uniaxial (along z-axis) strain have significant effects on the stability of magnetic order and orbital ordering. The pressure not only strengthens spin-fluctuations and lowers the transition temperature but also suppresses the orbital ordering. On the other hand, the tensile strain enhances the spin fluctuation and lowers Tc, whereas the compressive strain behaves the opposite way. Our findings suggest STM experiments or ultrafast experiments to measure the orbital ordering and the magnetism with/without pressure or strain.

## Method

First-principles electronic structure calculations for several magnetic phases of Sr_2_RuO_4_ with 4 formula unit (f.u.) in the unit cell (u.c.) are performed using the full-potential projected augmented wave method^44^ as implemented in the Vienna ab initio simulation package (VASP)^45^ based on the density functional theory (DFT). Both the local density approximation (LDA) and the LDA plus on-site Coulomb interaction U (LDA + *U*)^46^ method are used for the self-consistent calculations over the 12 × 12 × 6 k-point mesh in the Brillouin zone with the cutoff energy of 400 eV for the plane waves. On-site Coulomb energy $U=3.5$ eV and exchange parameter $J=0.6$ eV^47^ are used for Ru ions to explore the correlation effects in 4d electrons. The spin-orbital coupling (SOC) is also taken into account to study the influence in orbital and magnetic properties. For each magnetic phase, the lattice structures are fully optimized using the conjugate-gradient algorithm within the convergence criterion of 10^−4^ eV. The surface orbital ordering properties are also studied using the LDA + *U* method in the slab calculations with the slab thickness of 3~4 u.c. (5~7 RuO_2_ layer) for both the SrO- and RuO_2_-terminated surfaces using 5 × 5 × 1 k-points over the 2D Brillouin zone after the lattice optimization.

## Data Availability

All data generated or analysed during this study are included in this published article.
